# Discussion on Hysteria

**Published:** 1891-11

**Authors:** 


DISCUSSION.

In the discussion Dr. Poynor said that he was disappointed in that he expected a paper from an experimental standpoint. In the disease the will power yields to the emotional power of the individual. It was increasing, and would continue to do so on account of the rapid development of the emotional faculties. He recommended highly the suggestion of cultivating in every way the mothers of the future. He never noticed hysteria among the negroes during slavery.

Dr. Denton commended the paper, but took exception to the statement that hysteria is hereditary. He did not think the history of the human race would bear out the assertion. He had seen it among slaves but very rarely. It was merely a symptom and depended, in his opinion, like every neurosis, on a physical cause, and the only rational treatment was to seek for and remove that cause. In his experience he had met with no case of hystero-epilepsy in which sufficient reasons for the symptoms were not found.

Dr. Christian heartily endorsed the paper and concurred in the opinion as to heredity. He did not believe that the disease always depends on a physical cause, but thought that moral conditions often played a prominent part. Most good was to be obtained from strong mental impressions, change of scenery and associations, stimulating the will power and making the patient understand that she could control the paroxysm.

Dr. Leake said she did not desire to give the impression that the disease was hereditary like phthisis. She spoke of how the invention of labor-saving machines, by taking away employment from women predisposed to emotional development.

